# Chemosensory Impairments and Their Impact on Nutrition in Parkinson’s Disease: A Narrative Literature Review

**DOI:** 10.3390/nu17040671

**Published:** 2025-02-13

**Authors:** Sonila Alia, Elisa Andrenelli, Alice Di Paolo, Valentina Membrino, Laura Mazzanti, Marianna Capecci, Arianna Vignini, Mara Fabri, Maria Gabriella Ceravolo

**Affiliations:** 1 Department of Clinical Sciences, Università Politecnica delle Marche, 60126 Ancona, Italy; s.alia@pm.univpm.it (S.A.); a.dipaolo@pm.univpm.it (A.D.P.); v.membrino@pm.univpm.it (V.M.); 2 Department of Experimental and Clinical Medicine, Neurorehabilitation Clinic, Università Politecnica delle Marche, 60126 Ancona, Italy; e.andrenelli@univpm.it (E.A.); m.g.ceravolo@univpm.it (M.G.C.); 3 Fondazione Salesi, Ospedale G. Salesi, 60123 Ancona, Italy; mazzanti.laura@gmail.com; 4 Department of Life and Environmental Sciences, Università Politecnica delle Marche, 60131 Ancona, Italy; m.fabri@univpm.it

**Keywords:** Parkinson’s disease, smell, taste, nutrition

## Abstract

Parkinson’s disease (PD) is a neurological disorder characterized by heterogeneous symptomatology, in which the classical motor features of Parkinsonism are associated with clinically significant non-motor symptoms. Olfactory alteration, as a manifestation of PD’s premotor or prodromal phase, is well known. These impairments can lead to malnutrition, decreased appetite, and depression, thereby worsening patients’ quality of life. However, only a few studies clarify the mechanisms, characteristics, and clinical diagnostic and therapeutic implications of impaired taste perception. Moreover, unlike most motor features of PD, non-motor symptoms often have limited treatment options or responses. The purpose of this review is to collate and describe all relevant studies on taste and smell alterations in patients with PD and how these alterations could affect nutritional status. Our search aimed to identify English-language research articles and reviews published in peer-reviewed journals over the past two decades (2004–2024), while also including older foundational studies when relevant. Several studies show that hyposmia in PD worsens over time, potentially linked to structural changes in the brain’s basal ganglia and piriform cortex. Severe hyposmia is also associated with a higher risk of dementia in PD patients and can negatively influence quality of life, affecting social interactions and nutrition. Regarding taste perception, recent studies have suggested that hypogeusia may occur even in the prodromal stage of PD, such as in patients with REM sleep disorder, although the exact mechanisms remain unclear. Additionally, research has explored the role of bitter taste receptors and their possible involvement in inflammation and α-synuclein misfolding, suggesting a link between taste dysfunction and immune system changes in PD. Attention was then focused on the gut microbiota’s link to the central nervous system and its contribution to gustatory dysfunctions, as well as how the nasal microbiome influences PD progression by altering the olfactory system. Nowadays, the primary role of a correct diet in the overall treatment of PD patients is becoming increasingly important for practitioners. Diet should be included among the available aids to counteract some aspects of the pathology itself. For all these reasons, it is also crucial to determine whether these chemosensory impairments could serve as disease markers, helping to better understand the underlying mechanisms of the disease.

## 1. Introduction

### 1.1. Epidemiology and Symptoms

Parkinson disease (PD) is a neurodegenerative disease described for the first time in 1817 by the British physician, paleontologist, and geologist James Parkinson in his famous treatise “An Essay on the Shaking Palsy” [1]. In recent years, PD has undergone fast growth among neurological disorders, and it has become one of the leading causes of disability worldwide, affecting more than 10 million people worldwide [2,3]. The incidence of PD ranges from 5/100,000 to over 35/100,000 new cases per year, and it increases 5- to 10-fold in the sixth to ninth decade of life [4]. In addition to age, which remains the most important risk factor for the disease, and the male gender, environmental factors, including certain pesticides, and living in rural areas or having agricultural occupations have also been associated with PD risk [5]. β-adrenergic receptor antagonists have been linked to an increased risk of PD, while β-2 adrenergic receptor agonists appear to reduce it. On the other hand, there is an inverse association between the risk of PD and cigarette smoking, coffee consumption, statins, and calcium antagonist use, while conflicting evidence is available on the use of nonsteroidal anti-inflammatory drugs and altered levels of uric acid or gout [5]. The crucial pathological feature of PD is the loss of dopaminergic neurons within the Substantia nigra pars compacta (SNpc); the most deeply affected area is typically the ventrolateral level, which contains neurons projecting to the dorsal putamen. Clinical/pathological correlation studies have shown that the dopaminergic neuronal loss within this area is probably the cause of many of the motor symptoms, particularly bradykinesia and rigidity. Furthermore, neuronal loss also occurs in other brain regions such as the locus coeruleus, the basal nucleus of Meynert, the pedunculopontine nucleus, the raphe nucleus, the dorsal motor nucleus of the vagus, the amygdala, and the hypothalamus [6]. Another distinctive anatomo-pathological feature of PD is the Lewy pathology caused by the aggregation of misfolded proteins, made up of insoluble α-synuclein aggregates responsible for the formation of Lewy bodies (LBs) and Lewy neurites (LNs). Lewy pathology can also be found in the spinal cord and peripheral nervous system, including the vagus nerve, sympathetic ganglia, cardiac plexus, enteric nervous system, salivary glands, adrenal medulla, cutaneous nerves, and sciatic nerve [6].

### 1.2. Non-Motor Symptoms

Non-motor symptoms may play an important role, as they may constitute early manifestations of PD preceding the onset of motor disturbances and including a wide range of manifestations, ranging from hyposmia to constipation, rapid eye movement (REM) sleep disturbance, pain, depression, etc. Therefore, the study of these symptoms is important, as they contribute to the altered QoL of patients, especially with the progression of the disease, and constitute important therapeutic targets [1,7].

The altered olfactory perception often precedes the onset of motor symptoms by at least 4 years, and it constitutes one of the early clinical markers of PD. Equally important is taste perception, the other chemosensory system of the body, which is closely related to the sense of smell. Alterations in taste perception have also been described in PD, but there are still few studies able to clarify the mechanism responsible for such alteration, as well as the clinical, diagnostic, and therapeutic implications [8,9]. Loss of smell is associated with accumulation of α-synuclein in the olfactory bulb, amygdala, and other limbic structures, which appears to be a central mechanism in PD neurodegeneration. Alterations in smell and taste reflect the impairment of dopaminergic pathways and cholinergic neuronal networks, providing a model to study neurodegenerative progression [10]. Both the loss of smell and taste can negatively influence appetite and food preferences in PD patients, increasing the risk of malnutrition and weight loss. These sensory deficits can have a major impact and also contribute to depression, anxiety, and social isolation, compounding the overall impact of the disease on QoL [10].

Since olfactory impairment is significantly more severe in patients with PD than in other neurodegenerative diseases, such as Alzheimer’s (AD), this could make olfactory assessment a useful tool to differentiate PD from other neurological conditions [11]. Understanding olfactory and gustatory alterations can lead to diet optimization to improve QoL [11].

The present review is aimed at highlighting recent findings on the alterations of olfactory and gustatory sensitivity, which may represent a serious burden in PD patients since they are associated with an increased risk of malnutrition with a consequent decrease in appetite and depression, negatively influencing their QoL [12].

## 2. Methods

Given the narrative nature of this review, a literature search was conducted with the purpose of providing a comprehensive overview of current knowledge on the chemosensory alteration in Parkinson’s disease. A comprehensive literature search was conducted using multiple databases: PubMed, Scopus, and Web of Science. The search aimed to identify significant English research articles and reviews published in peer-reviewed journals over the last 2 decades (2004–2024), although older introductory studies were also included when relevant. This time frame was chosen to ensure the review focused on the most current and relevant research, which reflects the recently emerging trends and findings in the field. The attention was focused on both in vivo human and animal studies to ensure clinical relevance and exclude in vitro research studies or research without significant outcomes related to impairment in taste and smell in PD. The search terms were carefully chosen to include an extensive range of research on the relationship between the alteration in taste and smell in PD. The following keywords and phrases, in different combinations, were used: “taste alteration”, “smell alteration”, “taste recognition” (including specific terms like sweet taste, sour taste, salty taste, umami taste, bitter taste), “taste impairment” (including specific terms like dysgeusia, ageusia, hypogeusia, hypergeusia, fantageusia), “smell impairment” (including specific terms like parosmia, dysosmia, anosmia), chemosensory disorders, chemosensory dysfunction, aging, and quality of life. To enhance the search process, some Boolean operators (AND, OR, and NOT) were also used. Inclusion criteria included studies that explored the effects of taste and smell alteration in PD, discussed mechanisms underlying chemosensory impairment on PD, or provided clinical insights into QoL life modifications for PD patients. Exclusion criteria ruled out articles in languages other than English, studies published before 2024 unless considered seminal works crucial for background information, in vitro studies, and articles without significant clinical data. This search approach ensured an exhaustive and widespread review of the available literature, focusing on studies that offered clinical consequences and mechanistic insights into the relationship between taste and smell alteration and PD.

## 3. Parkinson’s Disease and Sensory Impairment

The chemosensory functions of taste and smell are closely related and play a vital role in both human and animal physiology. The first sensation perceived during a meal is smell, which is mediated by the volatile molecules released from food that bind to receptors in the olfactory mucosa. Subsequently, gustatory perception occurs when food is introduced into the oral cavity, where it interacts with specific receptor cells. This is followed by a second olfactory and gustatory perception through the nasopharynx and larynx [12,13,14,15].

### 3.1. Anatomy of the Olfactory System

The olfactory system is responsible for identifying and processing olfactory signals. In mammals, olfactory detection begins in the nasal olfactory structures and proceeds to the olfactory bulb, the first central relay. This telencephalic structure is the first site where sensory stimuli and environmental pathogens come into a direct contact, which may impact its specific neuronal cells. The olfactory epithelium, containing the receptors, covers the lamina cribrosa, the superior nasal septum, and the middle and superior turbinates, with variable distribution among individuals. The olfactory receptor cells, which are the primary sensory neurons with bipolar morphology, are interspersed with support cells. Basal stem cells, located on the basal surface of the epithelium, serve as progenitors that differentiate into new bipolar cells [12,16,17,18]. The short dendritic process of the bipolar olfactory cells at the mucosal end forms a bulb-shaped expansion, from which the olfactory cilia emerge. At the apex of the cilia, receptor proteins are present, where the inhaled odorous molecules bind and initiate olfactory transduction. This binding activates a G protein-coupled second messenger pathway, ultimately leading to the generation of action potentials in the primary neurons. In the olfactory bulb, second-order sensory neurons and interneurons are found; the former include small tufted cells and large mitral cells, whose axons project through the olfactory tract to secondary olfactory areas. The latter include periglomerular and the granular cells. Axons of the olfactory system project directly to cortical structures. The olfactory tract sends information to the piriform cortex, the main center of olfactory discrimination, which is necessary for the cognitive awareness of smell. Projections also reach two regions of the limbic system, which process the affective, mnemonic, and hedonistic components of olfactory stimuli—the cortical nucleus of the amygdala and the entorhinal area—which then project to the hippocampus.

The axons of mitral and tufted cells projects, through the olfactory tract, to the anterior olfactory nucleus, and the olfactory tubercle, which in turn projects to the dorsomedial nucleus of the thalamus. From there, it sends information to the orbitofrontal cortex, whose lesion compromises the ability to distinguish odors. Therefore, the thalamus acts as an olfactory relay between the entorhinal and orbitofrontal cortex, the latter being responsible for integrating olfactory information with that from other sensory receptors [12]. The olfactory system has remarkable sensitivity and discriminative capacity. Olfactory acuity varies significantly among healthy individuals. Olfactory dysfunction affects about 1% of subjects under the age of 60 and more than 50% of older individuals, reaching 75% after the age of 80, especially in men. It commonly involves the perception of pleasant smells, regardless of culture. Recent studies indicate that the prevalence of olfactory dysfunction ranges from 9% to 24% in the general adult population [12,19]. Smell disorders include anosmia (total or specific to a single odorous substance or a group of related substances), hyposmia, hyperosmia, dysosmia, phantosmia, agnosia, cacosmia, and parosmia. The underlying mechanisms may involve pathological conditions that interfere with the access of the odorous substance to the olfactory neuroepithelium, constituting a transport deficit, lesions in the receptor region leading to sensory deficits, or damage affecting the central olfactory pathways, resulting in neural deficit [12].

### 3.2. Smell Alterations in Parkinson’s Disease

Olfactory dysfunction is a prominent non-motor feature of Parkinson’s disease (PD), first described in 1975, and affects over 90% of patients. It often manifests years before motor symptoms and serves as an early indicator of PD [20]. A study by Postuma et al. showed that individuals with reduced smell and REM sleep disorders have a 65% chance of developing neurodegenerative disease, compared to only 14% in those with a normal sense of smell [21]. Over time, hyposmia worsens in PD patients, as demonstrated in a 4-year study involving olfactory tests and MRI. The study revealed that basal ganglia volume reduction correlated with sensory dysfunction in PD patients, although the progression of hyposmia was not significantly faster than in controls [22]. Research on mice has provided insights into the mechanisms behind hyposmia in PD. Neuroinflammation in the piriform cortex, caused by toxins mimicking premotor PD, resulted in damage to the neural network [23]. This was accompanied by a reduction in noradrenergic and dopaminergic cells and decreased calbindin and VIP in interneurons. However, Exendin-4 (EX-4) therapy, which activates GLP-1R, was able to prevent much of this damage [23].

Imaging studies in PD patients with varying degrees of hyposmia revealed that severe hyposmia is associated with a higher risk of cognitive decline and dementia [24]. Functional MRI and voxel-based morphometry (VBM) studies showed reduced connectivity between the amygdala and areas like the lower parietal lobe and fusiform gyrus in patients with severe hyposmia. However, compensatory connectivity in other brain regions was observed in patients with mild or no hyposmia, indicating potential mechanisms for preserving cognitive function [24].

Polymorphisms in genes that code for membrane receptors or odorant-binding proteins (OBPs)—carrier proteins responsible for transporting odor molecules to receptor sites—have been identified as factors contributing to functional variations in olfactory ability. Recently, the rs2590498 (A/G) polymorphism in the OBPIIa gene has been shown to influence retronasal and olfactory perception. Individuals with the A allele generally exhibit higher sensitivity to odors compared to those with the G allele. Bioinformatics analysis suggests that this mutation reduces OBPIIa protein expression in the olfactory epithelium [25]. This polymorphism also affects olfactory performance in women with PD. Specifically, women with PD who carry two sensitive alleles (AA) perform better in olfactory tests compared to women with at least one G allele and all men with PD. Notably, the olfactory scores of women with the AA genotype and PD are comparable to those of healthy controls. These findings indicate that the AA homozygous condition helps preserve olfactory function in women with PD, whereas this protective effect is absent in men. The OBPIIa locus could therefore serve as a molecular marker to identify the risk of olfactory deficits in women with PD [25].

PD is not the only neurodegenerative disorder associated with anosmia. Studies have demonstrated significant olfactory impairment in patients with AD as well [26]. In the early stages of AD, brain pathology can lead to changes in olfactory perception, offering valuable insights that may allow for use as an early biomarker [27,28].

Additionally, in the subsequent sections addressing taste alterations, we will examine how shifts in the gut microbiota may influence the central nervous system, thereby contributing to the onset of gustatory dysfunctions [29]. Concerning olfactory dysfunction, the nasal microbial community plays a critical role in the progression of PD, as evidenced by a reduction in the relative abundance of non-inflammatory bacteria, such as *Blautia wexlerae*, *Lachnospira pectinoschiza*, and *Propionibacterium humerusii*, within the nasal sinus cavity of PD patients [30].

### 3.3. Anatomy and Function of the Taste System

The gustatory function plays an important nutritional and social role, improves QoL, and regulates digestive and absorptive processes [31]. The perception of fundamental tastes (salty, sour, sweet, bitter, and umami) originates in the tongue and other gustatory tissues. Each taste is evoked by compounds of a different chemical nature and mediated by distinct mechanisms of signal uptake and transduction [32]. Taste sensitivity can be influenced by the concentration of the substance, gender (i.e., men generally perceive sweet and salty tastes less strongly, while their perception of sour tastes is greater), and age, as taste sensitivity tends to decrease and change with age [13,33]. Recently, it has been shown that taste sensitivity can also be affected by the body mass index (BMI) [34] and glycaemia [35,36,37].

The gustatory system provides information about ingested food, allowing for the evaluation of its identity, energy and/or nutritional content, potential danger, concentration, and unpleasantness. The ability to taste food depends on the presence of gustatory receptor cells, specialized cells located in the oral cavity and stimulated by chemicals in food. These receptor cells are found on the surface of the tongue, palate, epiglottis, pharynx, larynx, and the proximal part of the esophagus, and are grouped in rounded structures called taste buds. Taste buds consist of taste sensory cells, basal cells, and support cells. The microvilli on the top of the taste sensory cells are in communication with the oral cavity through the gustatory pore. Each taste bud contains between 50 and 150 cells and is grouped into specialized structures called papillae, which have four different morphologies: fungiform, foliate, circumvallate, and filiform (the latter seems to be more sensitive to touch and temperature rather than taste) [13,18,32,38,39]. Gustatory information is transmitted to the CNS via the afferent fibers of three cranial nerves: (i) the facial nerve (VII cranial nerves), (ii) the glossopharyngeal nerve (IX), and (iii) the vagus nerve (X) [40].

The gustatory system can detect or “taste” a wide variety of chemical molecules, which are generally grouped into five fundamental tastes: sweet, bitter, sour, salty, and umami (or savory). According to recent studies, fat and water should also be considered basic tastes [41,42]. The sweet taste helps identify energy-rich nutrients and is evoked by the stereochemical configuration of sugars; the salty taste ensures an appropriate balance of electrolytes and is triggered by sodium ions; the sour and bitter tastes alert the body to the potential ingestion of irritating and poisonous substances, with sourness caused by hydrogen ions and bitterness caused by compounds containing nitrogen. A fifth fundamental gustatory stimulus, umami, was added in 1985, and is associated with monosodium glutamate (MSG), helping to detect amino acids, the building blocks of proteins, which are essential components of the body and diet [43,44,45,46]. Additionally, the fatty taste has been recognized as a fundamental taste in the past decade. Free fatty acids, produced by the rapid hydrolysis of ingested triglycerides mediated by the salivary lipase, serve as strong gustatory stimuli and are recognized by specific receptors located on the membrane of taste cells [40,41,42,43,44,45,46]. Even more recently, growing evidence from human and other animal studies suggests the existence of a taste modality responsive to water, which is thought to be mediated by aquaporins (AQPs), considered channels for water molecules [42].

### 3.4. Taste Alteration in Parkinson’s Disease

Taste disorders can range from a complete loss of taste (ageusia) to phantom or distorted tastes (e.g., bitter or metallic). These alterations can lead to severe consequences, including malnutrition, obesity, hypertension, and even death [34,47]. Taste disorders are categorized into six main types: hypogeusia (reduced taste sensitivity), hypergeusia (heightened sensitivity), dysgeusia (distorted taste), phantogeusia (phantom taste, which means perception of a certain taste even when no substance has been ingested), ageusia (loss of taste), and cacogeusia (unpleasant taste in the absence of food). They may result from various causes, such as autoimmune diseases, inflammation, neurodegenerative disorders like PD and AD, psychological issues, poor oral hygiene, surgery, chemotherapy, or natural aging [35,47,48,49]. Despite their prevalence, the mechanisms behind these disorders and their clinical implications remain poorly understood [8,9].

Although olfactory impairment is a well-known early symptom of PD, hypogeusia is less studied and has yielded controversial results [50,51,52,53]. PD patients experience dysgeusia across all basic tastes, which impacts their appetite and dietary choices. Since they show a preference for sweet, salty, and umami flavors, this can be utilized to tailor their diets in a way that may help enhance their nutritional status [54]. Another recent case–control study evaluated taste function in 44 patients with REM sleep disorder (a prodromal phase of PD), 19 PD patients, and 29 healthy controls [51]. Results showed reduced taste sensitivity in both REM sleep disorder and PD patients, suggesting that hypogeusia may occur in prodromal PD [51]. Importantly, no correlation was found between hypogeusia and smell impairment, highlighting that these sensory deficits may have distinct underlying mechanisms [8]. Taste sensitivity may also be influenced by sialorrhea (excessive salivation), common in late-stage PD [55]. Sialorrhea leads to swallowing difficulties, reduced QoL, social embarrassment, bacterial growth, and aspiration pneumonia [56]. Additionally, 9–22% of taste disorders are linked to medications, including dopaminergic drugs, which can modulate taste perception [57]. The bitter receptor identified as TAS2R38 (member of the T2R receptors) has been widely studied, as polymorphisms of the gene give rise to variants of the receptor with a different affinity for the stimulus. Bitter taste receptors T2R encodes seven transmembrane G-protein coupled receptors that were originally identified on the tongue, but both the nasal and bronchial airways and the intestinal mucosa express multiple T2R isoforms (T2R4, T2R14, T2R16, and T2R38). Several authors have proposed an interaction between T2R signaling and toll-like receptors (TLRs). TLRs have been implicated in innate immunity, and their dysregulated activity is reported to play a role in α-synucleinopathy. Further studies are needed to confirm whether the altered T2R observed in PD may play a specific role in inflammatory mechanisms associated with the initiation of α-synuclein cascade misfolding, which modulates innate immunity via TLR/T2R signaling [58,59]. In addition to its gustatory role, TAS2R38 is expressed in the gut epithelium, where it interacts with the microbiota and modulates immune responses. In PD, the non-taster TAS2R38 variant is associated with decreased microbial diversity, particularly with a reduction in the *Clostridium genus*. This decrease in diversity may impair protective signaling pathways that help maintain gut homeostasis [29]. The associated alterations in gut microbiota may contribute to PD progression via mechanisms involving the gut–brain axis. But further investigation is needed to clarify these interactions and their potential role in PD management [29].

## 4. Importance of Nutrition in Parkinson’s Disease

It is very important to study the alterations of olfactory and gustatory sensitivity, which can represent serious disorders as they influence the QoL of patients with PD. PD patients often lose interest in cooking, develop a poor appetite, which can lead to weight loss with consequent malnutrition, prefer sweet and salty foods (increasing the risk of diabetes and hypertension), and experience reduced social interactions. These changes have been increasingly studied over the last 15 years [12,60].

Many years after the initial description of PD, more attention is turning to diet and nutrition with regard to the progression of the disease [61]. Therefore, it is essential to focus attention on the development of nutritional guidelines for the screening, as well as the evaluation and management, of PD. Although both low and high body weight have been reported as risk factors for PD, weight loss negatively affects disease severity and increases the risk of dyskinesias as the disease progresses, which in turn negatively impacts QoL and increases mortality [62]. Alterations in sensory perception significantly impact food choices and intake. Given the high prevalence of hyposmia up to 90% [63] and the potential influence of hypogeusia, a reduction in smell and/or taste could worsen the risk of malnutrition in PD patients [53].

It should also be emphasized that drug therapy must be accompanied by correct nutritional surveillance. Barichella and colleagues [64] conducted a large case–control study involving 600 PD patients and an equal number of healthy controls, examining the relationship between dietary habits and several PD characteristics, including body weight, energy balance, constipation, levodopa therapy, and motor complications. They found that PD patients, despite consuming more food than controls, had a lower BMI, which was inversely related to disease duration, severity, and levodopa-related motor complications. Psychological factors (e.g., depression, social withdrawal) and physical issues (e.g., dysphagia, dyskinesia) increased the risk of malnutrition. Additionally, reduced water intake and higher protein consumption interfered with levodopa effectiveness due to competition with amino acids during absorption. The management of protein intake and constipation treatment should be integral to PD care. Maintaining proper energy intake is crucial for sustaining nutritional status, optimizing levodopa therapy, and minimizing motor complications. Recommendations include limiting protein intake to 0.8 g/kg of ideal body weight, drinking 2 L of water daily, and moderating fat consumption to avoid delaying gastric emptying and promoting levodopa degradation [64].

Among the factors associated with weight change, advanced age, disease severity, imbalance in food intake, cognitive disorders but also impaired olfactory and taste sensitivity could be mentioned [62,65,66]. Gruber et al. [67] conducted a cross-sectional study with 92 hospitalized elderly PD patients (mean age 73.6 ± 6.7 years, mean disease duration 7.9 years, no dementia), assessing nutritional status using the Mini Nutritional Assessment (MNA). They found that half of the patients were malnourished or at risk of malnutrition. Malnutrition was mainly linked to poor emotional well-being, suggesting that addressing depression and anxiety, alongside dietary and physical activity interventions, could improve nutritional status. Early identification of patients at risk for weight loss (e.g., anosmia, lower initial body weight, and increased risk of dyskinesia) should prompt strategies to prevent further weight loss [62]. Nutrition plays a crucial role in the health of PD patients, as symptoms like dysphagia, tremors, and muscle stiffness can impact food intake. Additionally, changes in taste sensitivity can influence food preferences, making meal enjoyment and nutritional intake more interesting. With appropriate dietary adjustments and ongoing healthcare support, these challenges can be managed more effectively [62]. For instance, patients should be advised on alternative sources of protein (such as nuts, beans, and seeds) and calcium (such as leafy greens, almonds, and tofu), as individuals with PD may be at risk of deficiencies in these nutrients.

Therefore, it is essential for practitioners to monitor patients with PD for signs of malnutrition during regular follow-up visits, especially patients with high-risk characteristics. Moreover, a comprehensive treatment plan for PD should include regular nutritional assessments and consultations with a dietitian to develop an individualized dietary regimen. Addressing both sensory impairments and nutritional needs can help prevent malnutrition and improve the overall health and QoL of patients.

## 5. Conclusions

Parkinson’s disease is complex in its clinical expression and management. Non-motor symptoms, such as loss of smell, are present from the premotor or prodromal stage and are associated with reduced health-related QoL. Identifying disease markers that allow for disease-modifying therapy administration is necessary to better understand the mechanisms underlying the pathology and to operate an appropriate approach for each patient. While olfactory dysfunction is conspicuously clear in PD and can be present many years before the onset of the disease, less is known about taste sensitivity in PD. The ability to taste is crucial to perform numerous important functions in humans, interacting with other sensory capacities, the gastrointestinal system, and the activities carried out by numerous brain areas. The most recent attention paid to the taste function has also highlighted its possible alterations, which can develop in physiological and pathological conditions.

In conclusion, as part of a comprehensive treatment plan for PD, it is important to incorporate regular nutritional assessments and consultations with a dietitian to develop a tailored dietary regimen. This approach can help address and prevent nutritional deficiencies, due to either smell and/or taste impairment, ensuring that patients receive the necessary support to maintain their health and QoL.

## Data Availability

No new data were created or analyzed in this study. Data sharing is not applicable to this article.
